# Redox proteomic study of *Bacillus cereus* thiol proteome during fermentative anaerobic growth

**DOI:** 10.1186/s12864-021-07962-y

**Published:** 2021-09-07

**Authors:** Fella Hamitouche, Jean-Charles Gaillard, Philippe Schmitt, Jean Armengaud, Catherine Duport, Luc Dedieu

**Affiliations:** 1 Avignon Université, INRAE, UMR SQPOV, Site Agroparc, F-84914 Avignon Cedex 9, France; 2 Université Paris-Saclay, CEA, INRAE, Département Médicaments et Technologies pour la Santé (DMTS), SPI, 30200 Bagnols-sur-Cèze, France

**Keywords:** *Bacillus cereus*, Thiol proteome, Anaerobiosis, Fermentative growth, Differential labeling strategy

## Abstract

**Background:**

*Bacillus cereus* is a notorious foodborne pathogen, which can grow under anoxic conditions. Anoxic growth is supported by endogenous redox metabolism, for which the thiol redox proteome serves as an interface. Here, we studied the cysteine (Cys) proteome dynamics of *B. cereus* ATCC 14579 cells grown under fermentative anoxic conditions. We used a quantitative thiol trapping method combined with proteomics profiling.

**Results:**

In total, we identified 153 reactive Cys residues in 117 proteins participating in various cellular processes and metabolic pathways, including translation, carbohydrate metabolism, and stress response. Of these reactive Cys, 72 were detected as reduced Cys. The *B. cereus* Cys proteome evolved during growth both in terms of the number of reduced Cys and the Cys-containing proteins identified, reflecting its growth-phase-dependence. Interestingly, the reduced status of the *B. cereus* thiol proteome increased during growth, concomitantly to the decrease of extracellular oxidoreduction potential.

**Conclusions:**

Taken together, our data show that the *B. cereus* Cys proteome during unstressed fermentative anaerobic growth is a dynamic entity and provide an important foundation for future redox proteomic studies in *B. cereus* and other organisms.

**Supplementary Information:**

The online version contains supplementary material available at 10.1186/s12864-021-07962-y.

## Background

*Bacillus cereus* is a Gram positive bacterium, which is recognized as a major foodborne pathogen responsible for two types of syndrome: emetic and diarrheal [1]. As well as being a facultative anaerobe, *B. cereus* can adapt to a wide range of environmental conditions allowing it to multiply in a number of food products and in the human intestine [2, 3]. In these environments, *B. cereus* adapts its metabolism to variations in temperature [4], pH, oxygen level, and oxidoreduction potential (ORP) [2]. *B. cereus* uses aerobic respiratory pathways to grow under aerobiosis, and mixed acid fermentation pathways to grow under anaerobiosis in the absence of an external electron acceptor. The main product of fermentation is lactate, which is synthesized alongside acetate, ethanol, formate, succinate, and small amounts of 2, 3-butanediol [5]. *B. cereus* re-oxidizes the reducing equivalent NADH, generated by glucose catabolism from NAD^+^, through the respiratory chain under aerobiosis, and during the formation of end-products under anaerobiosis [6]. Thus, NAD^+^/ NADH is central to catabolism and energy supply, whereas the NADP^+^/ NADPH couple plays an important role in biosynthesis and detoxification of cells [7]. Consequently, both the NAD and NADP systems play major roles in redox homeostasis [8]. Low-molecular weight compounds, such as coenzyme A, free cysteines, and bacillithiol (BSH) are also major contributors to redox homeostasis [9], in particular through their regulation of the redox status of the two amino-acids containing thiol (SH) groups, methionine (Met) and cysteine (Cys). Together, Cys and Met fulfill important roles in cells thanks to their redox chemistry [10]. Although the two residues are subject to redox regulation, the thioether form of the Met sulfur atom is less reactive than the sulfhydryl (thiol, SH) form of the Cys sulfur atom [10].

The redox status of Cys SH in proteins contributes significantly to protein folding [11], metal binding [12], and regulating protein function [10]. Thus, oxidation of Cys SH groups can result in the formation of reversible modifications such as disulfide bridges (S-S), sulfenic acid (S-OH) and nitrosylation (−SNO) groups, and irreversible oxidations such as sulfinic (R-SO_2_H) and sulfonic acid (R-SO_3_H) species [10]. The set of proteins containing reversibly modified thiols is referred to as the thiol proteome [13], or redoxome [14].

Attempts to study the thiol proteome have taken several approaches. Due to their high lability, cysteine modifications are challenging to analyze. Methods to identify Cys redox modifications have progressed over the years, ranging from the estimation of SH groups using colorimetric tests [15], and monitoring protein cysteine oxidation by loss of reactivity with thiol reagents [16], to accurately identifying sites of thiol redox modifications in numerous proteins [17]. The differential thiol labeling method constituted a major advance in the depth of analysis possible. The first step involves irreversibly blocking reduced-Cys thiols with an alkylating agent, subsequently the oxidized thiols are reduced, and finally labeled with a different alkylating agent [17]. This method has been combined with 2D gel-based methods [18, 19] to identify several redox-sensitive proteins. However, this combination presented limitations, mainly in the identification of cysteines. Today, the combination of differential thiol labeling with tandem mass spectrometry is a powerful means to overcome the limitations of previous methods and to provide broad coverage of the thiol proteome [14]. At the proteome level, reduced and oxidized Cys can be distinguished thanks to the use of different alkylating agents [13]. They can thus be quantified and the abundance of the proteins bearing these residues can be estimated [20].

Large-scale redox proteomic analysis has been used to investigate the relationships between the thiol proteome status in (i) cyanobacteria, focusing on photosynthesis and the response to nutrient limitation [21, 22], and (ii) *Clostridium difficile* [23], *Saccharomyces cerevisiae* [24], *Escherichia coli* [25, 26], and Firmicutes including *Bacillus subtilis* [27], to investigate responses to oxidative stress. These studies revealed that the thiol proteome modifies key biological processes through alterations to redox-sensitive proteins such as the elongation factor EF-Tu (Tuf), a key component of translation [28]; alcohol dehydrogenase (AdhA), which is involved in energy metabolism [23]; and alkyl hydroperoxide reductase (AhpC), a key player in defensive mechanisms [21].

Previous studies investigated the redox status of Met residues in unstressed *B. cereus* cells at both the cellular proteome and exoproteome levels [29, 30]. However, the thiol proteome of unstressed *B. cereus* cells has not yet been investigated. In this study, we used a thiol trapping method combined with shotgun proteomics analyses to decipher the *B. cereus* thiol proteome during fermentative anaerobic growth. Our results show that the *B. cereus* thiol proteome is growth-phase-dependent and contains higher number of reduced Cys in the later stages of growth.

## Results

### Labeling strategy for global analysis of the *B. cereus* thiol proteome

**Fig. 1:**
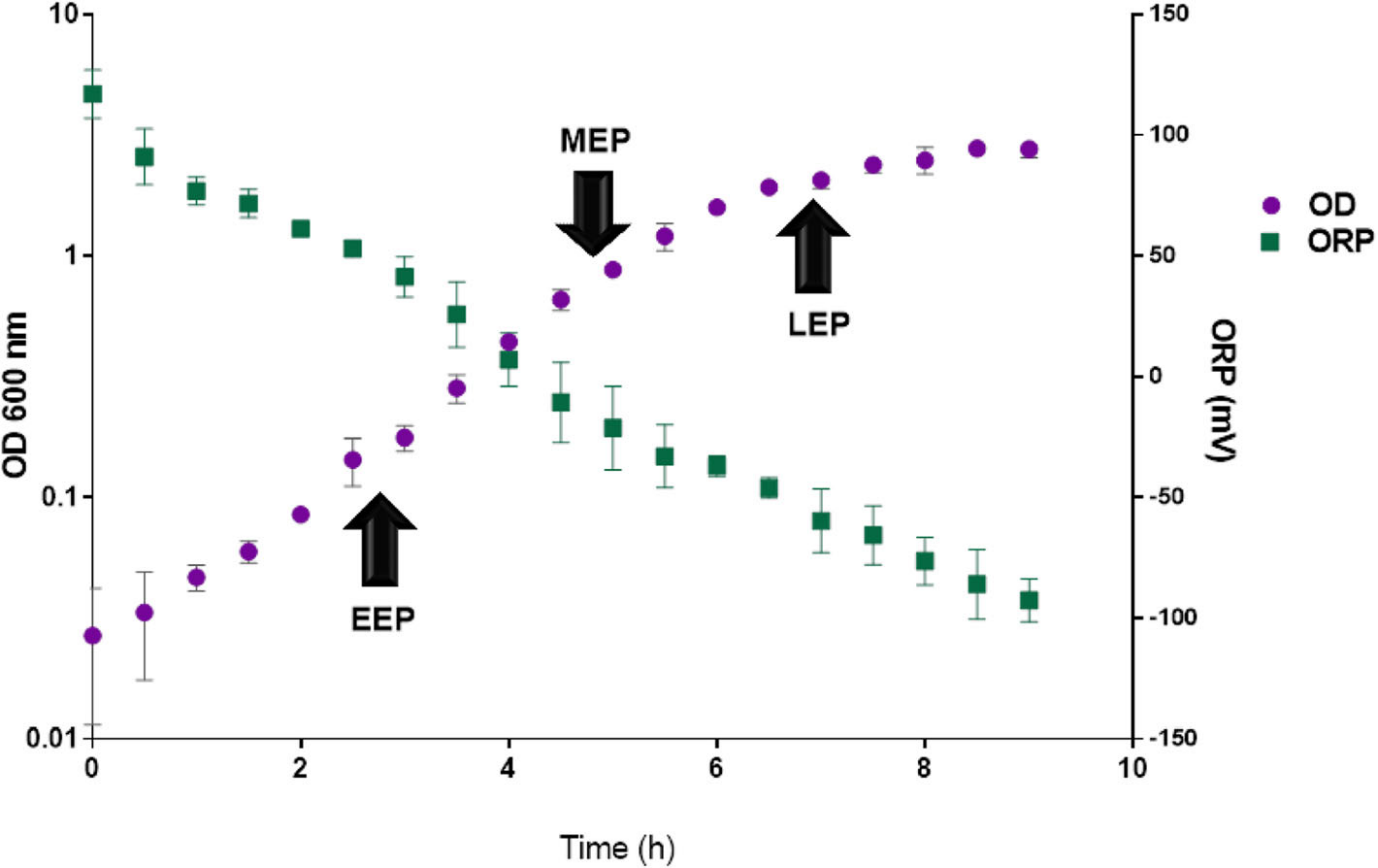
Growth curve and alterations to extracellular oxidoreduction potential (ORP) of *B. cereus* ATCC 14579 in regulated anoxic batch cultures. Circles represent optical densities at 600 nm (OD_600_) and squares correspond to extracellular ORP. Black arrows indicate cell harvesting, at early-exponential growth phase (EEP), mid-exponential growth phase (MEP), and late-exponential growth phase (LEP). Data correspond to mean values calculated for biological triplicates ± SD

Fermentative growth of *B. cereus* ATCC 14579 is illustrated in Fig. 1. It was accompanied by a decrease in extracellular ORP (ΔORP = 217 ± 20 mV). Culture samples were collected anaerobically, in TCA-containing vials at early- (EEP), mid- (MEP) and late-exponential (LEP) growth phases. Mixing with TCA resulted in immediate lysis of cells and precipitation of proteins, thus preserving the in vivo redox status of proteins containing Cysteine (Cys) residues [31].

**Fig. 2:**
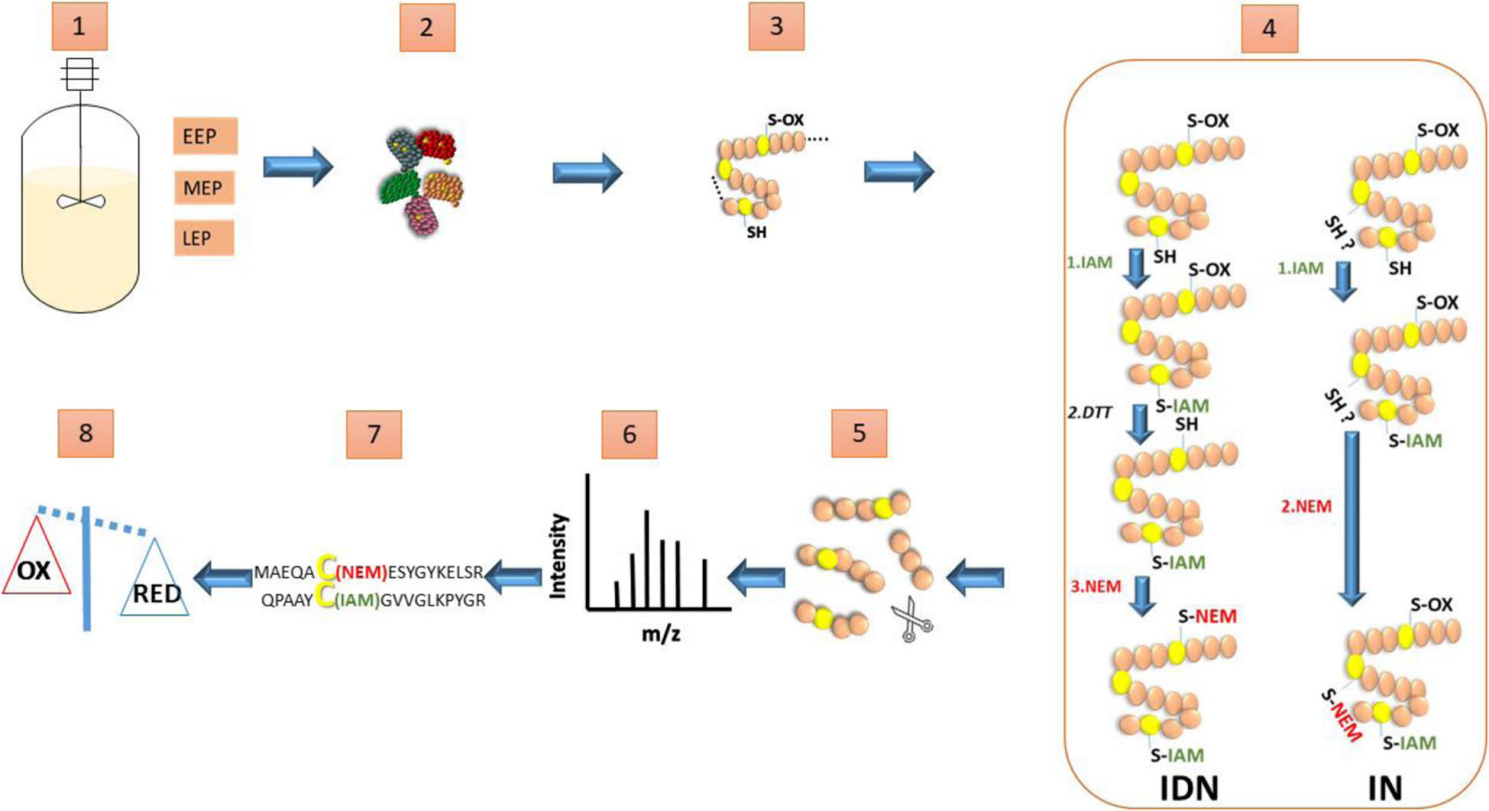
Schematic workflow used to investigate the *B. cereus* thiol proteome. 1/Samples were harvested from the bioreactor at early- (EEP), mid- (MEP) and late-exponential growth phase (LEP); 2/Proteins were extracted and thiol-disulfide exchanges were quenched by addition of TCA; 3/Denatured proteins were obtained; 4/Proteins with reactive Cys residues were labeled using two strategies. IDN) Reduced-Cys thiol (SH) were blocked with IAM; oxidized Cys thiols (S-OX) were then reduced with DTT and alkylated with NEM. IN) Reduced-Cys thiol were alkylated with IAM and remaining non-alkylated Cys thiols were blocked with NEM; 5/Proteins were digested with trypsin; 6/Peptides were analyzed by LC-MS/MS; 7/Modified Cys were identified; 8/ Proteins were identified and the thiol proteome profiled. TCA: trichloroacetic acid; IAM: iodoacetamide; DTT: dithiothreitol; NEM: N-ethylmaleimide

Reactive Cys residues were labeled using a three-step sequential strategy as previously described [32], and named IDN in our study (Fig. 2): First, iodoacetamide (IAM) was used to label and block free Cys thiol groups (SH). Then, reversibly-oxidized thiol (S-OX) were reduced by exposure to DTT, and finally the corresponding thiol groups were labeled using N-ethylmaleimide (NEM). The IN control omitted the DTT reduction step, and was used to assess how efficiently IAM blocked Cys SH. All proteins were digested with trypsin, and the resulting peptides were submitted to extensive LC-MS/MS analysis. A total of 354,684 peptides were identified from the 18 samples (3 cultures × 3 time-points × 2 labels), including 9531 Cys-containing peptides. A total of 20,952 ± 5265, 20,274 ± 4541, and 16,760 ± 2880 peptides were identified at EEP, MEP, and LEP, respectively. These values are not significantly different according to Student’s T-test.

**Fig. 3:**
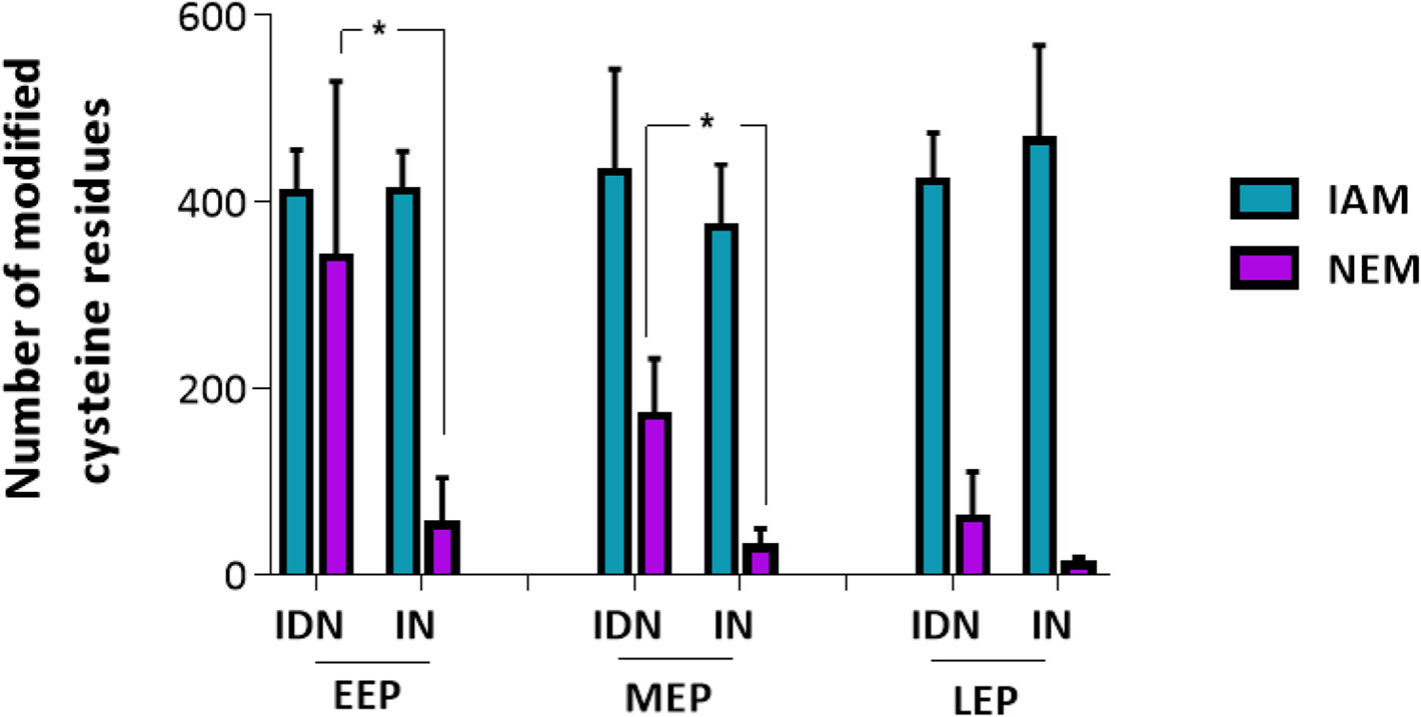
Labeling efficiency of IDN assay compared to IN control. IDN: reduced Cys were labeled with IAM and reversibly-oxidized Cys were reduced with DTT prior to labeling with NEM. IN: reduced Cys were first labeled with IAM and then with NEM without reduction between steps. The labeled Cys-containing peptides and Cys residues were quantified in samples harvested at early-exponential phase (EEP), mid-exponential phase (MEP) and late-exponential phase (LEP). Data correspond to mean values calculated for triplicates. Error bars show standard deviation. Paired Student t-test was performed to determine statistical significance (*: 0.01 < *p* < 0.05; **: 0.001 < *p* < 0.01; ***: *p* < 0.001)

The number of IAM-labeled Cys residues (IAMCys) was not significantly different in IDN-treated samples and IN controls, whatever the growth phase (Fig. 3). In contrast, as expected, the number of NEM- labeled Cys residues (NEMCys) detected was higher in IDN samples than in the IN control, for samples harvested during the EEP and MEP growth phases. Taken together, these results indicate that IAM alkylation stabilizes free sulfhydryl groups, but that a small number of Cys residues escape IAM alkylation and remain available for alkylation with NEM.

### *B. cereus* thiol proteome dynamics

NEMCys in IDN samples correspond to either in vivo oxidized Cys or insensitive IAM residues. To retain only in vivo oxidized Cys residues, we corrected the IDN data by subtracting NEMCys detected in the IN dataset from the list of NEMCys detected in the IDN dataset. The corrected IDN dataset (IDNc) is shown in Table S1.

**Fig. 4:**
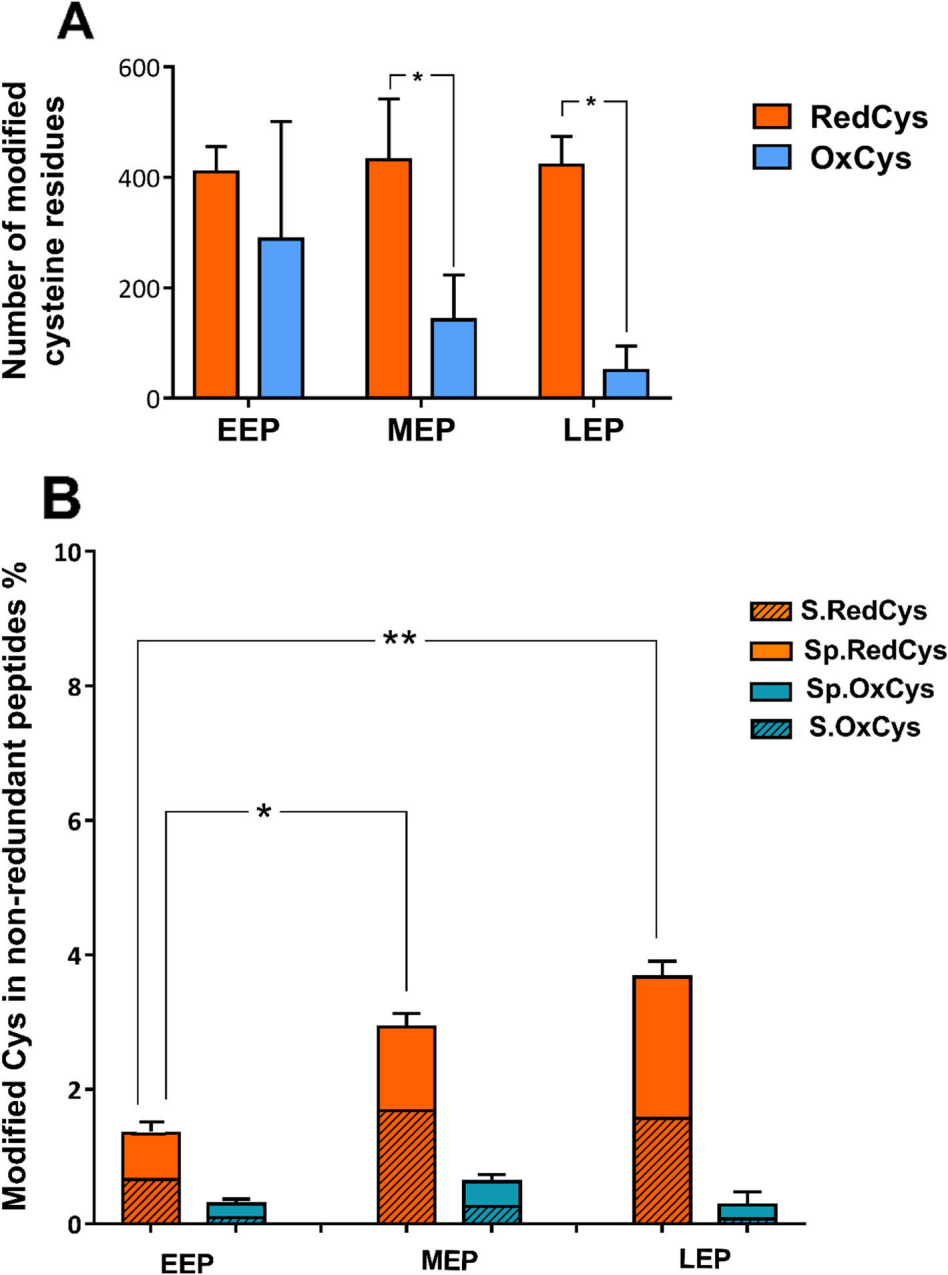
Proportion of reduced Cys (RedCys) and oxidized Cys (OxCys) residues identified in samples harvested at early-exponential, mid-exponential and late-exponential growth phases (EEP, MEP, and LEP, respectively). **A** Number of modified cysteines: RedCys and OxCys in samples. **B** Proportion of non-redundant RedCys and non-redundant OxCys residues. Sp.RedCys/Sp.Oxcys: reduced/oxidized cysteine residues that have been detected in only one growth stage (these residues are specific to a growth stage); S.RedCys/S.OxCys: reduced/oxidized cysteine residues that have been detected in two or three growth stages. Data correspond to mean values calculated for three replicates. Error bars show standard deviation. Paired Student t-test was performed to determine statistical significance (*:0.01 < *p* < 0.05; **: 0.001 < *p* < 0.01; ***: *p* < 0.001)

Analysis of the IDNc dataset shows that the numbers of RedCys (labeled with IAM) and OxCys (labeled with NEM after reduction) were significantly different at both MEP and LEP (Fig. 4A). In addition, the number of OxCys residues decreased during growth and the number of RedCys tend to increase as growth progresses. These data suggest that the thiol proteome evolved toward a highly reduced status as growth progressed. Due to trypsin missed cleavage and differences in abundance, some redox-sensitive Cys residues specified the same cysteine site. Removal of this redundancy resulted in a total of 110, 99 and 93 non-redundant (unique) redox-sensitive Cys residues at EEP, MEP and LEP respectively (Table 1). The number of the non-redundant redox-sensitive Cys residues decreased during growth due to an overall decrease in non-redundant peptide detection. This probably reflects the overall regulation of protein synthesis during growth [33]. However, the labeling efficiency remained stable (3 ± 0.1%, Table 1).

**Table 1:** Numbers of non-redundant peptides, Cys-containing peptides and Cys residues identified in all the three replicates at early-exponential phase (EEP), mid-exponential phase (MEP) and late-exponential phase (LEP)

	EEP	MEP	LEP
Non-redundant peptides	3673	3330	3087
Non-redundant Cys-peptides	109	95	88
Non-redundant Cys residues	110	99	93

**Fig. 5:**
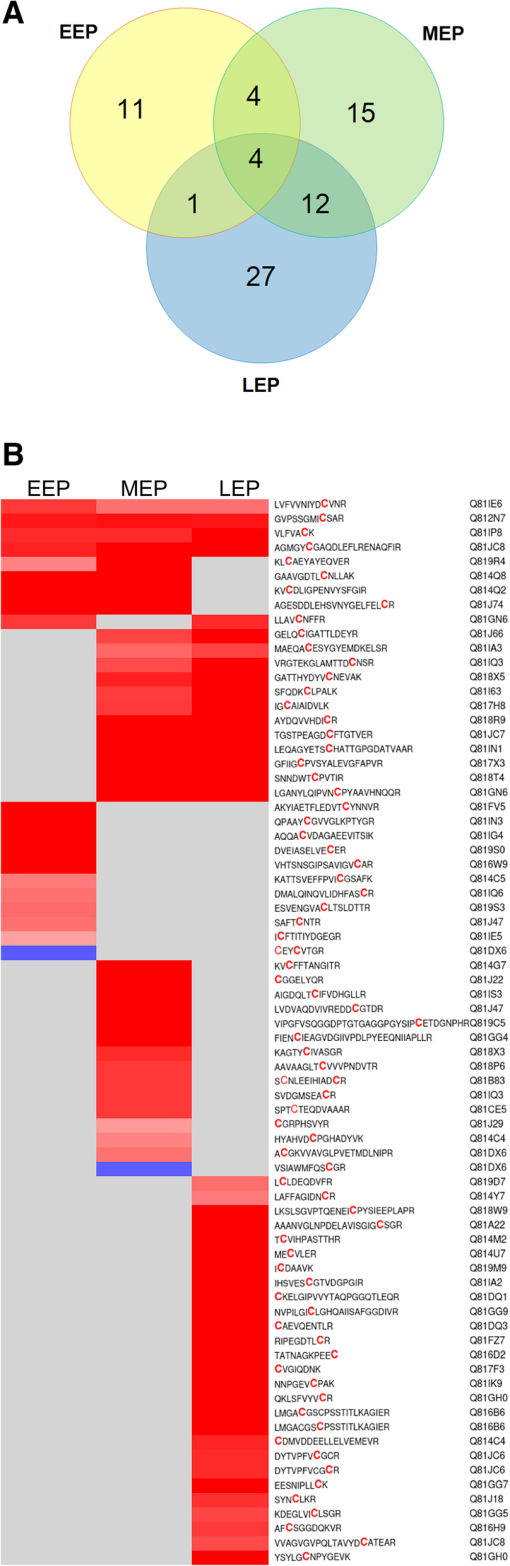
Growth-phase-distribution of the 74 Cys residues detected predominantly either as reduced Cys (RedCys) or as oxidized Cys (OxCys). **A** Venn diagram showing the growth-phase-distribution of the 74 Red/OxCys residues. **B** Heat map visualization of the 72 peptides containing the 74 Red/OxCys residues. EEP, early-exponential growth phase; MEP, mid-exponential growth phase; LEP, late-exponential growth phase. Colors represent the redox form that predominates. Blue: oxidized form; red: reduced form; gray: no significant difference

We compared the numbers of RedCys and OxCys of each non-redundant Cys residue. Based on this analysis, we identified 74 Cys residues for which the mean of RedCys number was significantly different from the mean of OxCys number (*p* < 0.05). Figure 5 shows their growth stage distribution (Fig. 5A), their RedCys vs OxCys distribution and the peptide sequences in which they were identified (Fig. 5B). The data indicate that (i) most of the 74 Cys residues were identified in only one growth phase and only two Cys residues had a higher number of OxCys compared to RedCys: one at EEP and the other at MEP. (ii) LEP samples supported the largest number of Cys residues, and (iii) the 74 Cys residues were contained in 72 non-redundant peptides.

If we consider all the 74 Cys residues, we do not observe any change in the total number of OxCys number during growth. In contrast, we observe a significant increase in the total number of RedCys at MEP and LEP, compared to EEP (Fig. 4B). This overall increase results from an increase in the number of RedCys residues identified in samples from at least two growth stages (hatched bars in Fig. 4B), and RedCys residues identified specifically in samples from only one growth stage (plain bars in Fig. 4B).

### Components of the *B. cereus* thiol proteome

**Fig. 6:**
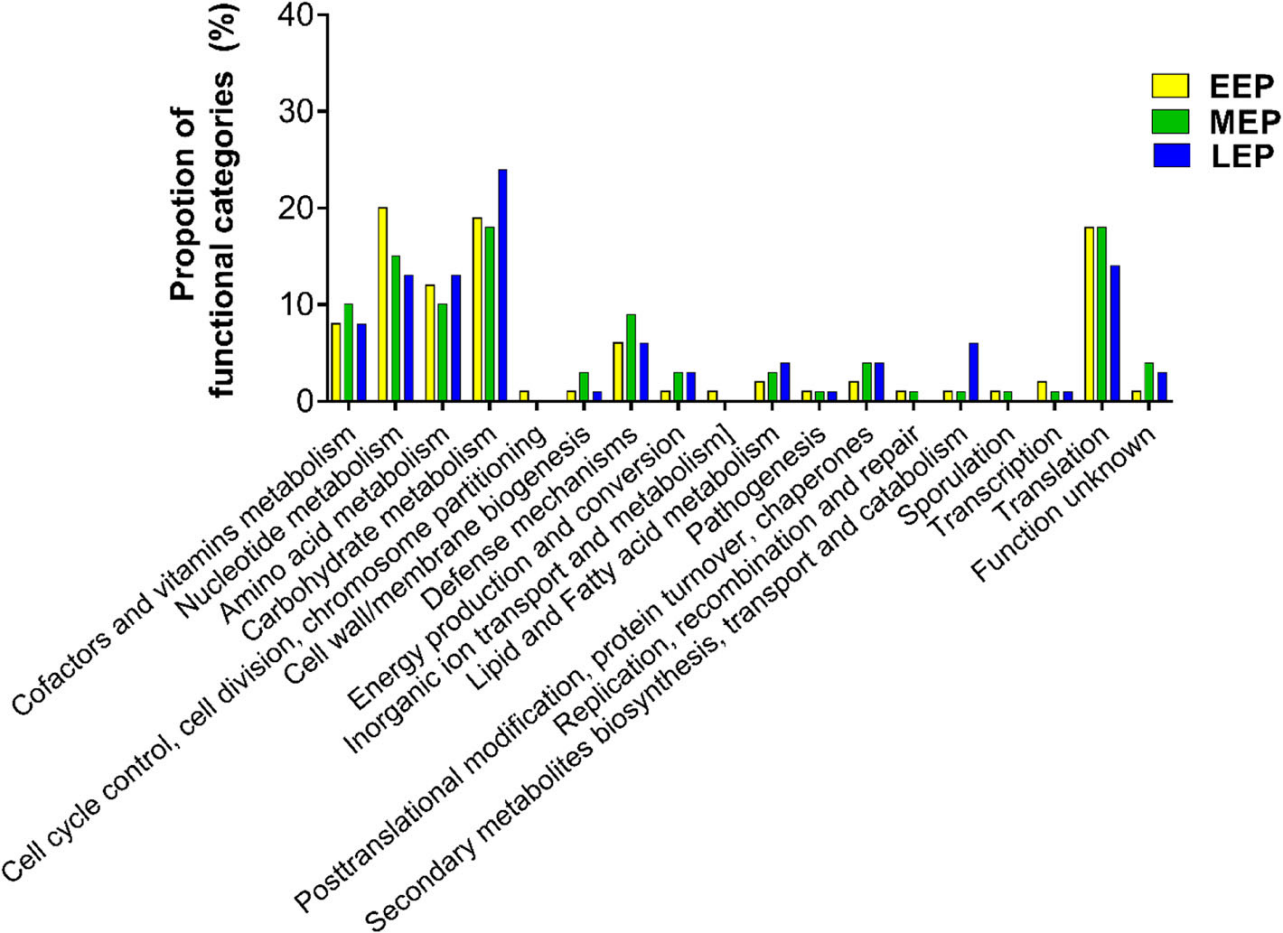
Functional categorization of reactive Cys-containing proteins at early (EEP), mid (MEP) and late (LEP) exponential growth stages

According to our results, the *B. cereus* thiol proteome includes 153 reactive Cys-containing peptides, of which 70 were detected mainly in the reduced form, and 2 in the oxidized form. We assigned these 153 peptides to their corresponding proteins. The resulting set consisted of 117 proteins (Table S2), of which 64 contained the 74 Cys residues identified above (Table 2). These proteins were mainly involved in translation, amino acid, nucleotide and carbohydrate metabolism, as well as defense mechanisms, whatever the growth stage (Fig. 6). Proteins related to carbohydrate metabolism include the glycolytic enzymes phosphofructokinase PfkA (Q817F3) and phosphoenolpyruvate PtsI (Q819D7), the fermentative enzyme pyruvate formate-lyase PflA (Q81IA2), and alcohol dehydrogenase (AdhA, Q81DX6), which also plays an important role in fermentative metabolism. Three distinct modified cysteines were identified in AdhA: Cys^255^ was mainly detected as RedCys at MEP, whereas the two others (Cys^97^ at EEP and Cys^91^ at MEP) were mainly detected as OxCys.

**Table 2:** Cys-proteins harboring specific cysteines detected as RedCys or as OxCys

	Cys position	Protein	Description	Log_**2**_(FC)	
				EEP/LEP	MEP/LEP
**Cys proteins in EEP,MEP,LEP**					
**Nucleotide metabolism**					
Q81IP8	381	PurD	Phosphoribosylamine-glycine ligase	NS	NS
Q81JC8	446	GuaB	Inosine-5′-monophosphate dehydrogenase	NS	NS
**Defense mechanisms**					
Q81IE6	123	TerD	Tellurium resistance protein	NS	NS
**Translation**					
Q812N7	170	PheT_1	Phenylalanine-tRNA ligase beta subunit	NS	NS
**Cys proteins in EEP,MEP**					
**Cofactors and vitamins metabolism**					
Q81J74	60		7,8-dihydroneopterin aldolase	NS	NS
**Translation**					
Q819R4	124	IleS1	Isoleucine-tRNA ligase 1	NS	NS
Q814Q8	153	ArgRS	Arginine-tRNA ligase	NS	NS
**Amino acid metabolism**					
Q814Q2	159	SpeB	Agmatinase	NS	NS
**Cys proteins in EEP,LEP**					
**Defense mechanisms**					
Q81GN6	458	Kat	Catalase	−2,46	−1,93
**Cys proteins in MEP,LEP**					
**Nucleotide metabolism**					
Q817X3	64	Apt	Adenine phosphoribosyltransferase	NS	NS
Q81IQ3	464	PurL	Phosphoribosylformylglycinamidine synthase subunit	NS	NS
**Carbohydrate metabolism**					
Q818T4	111		2-oxoisovalerate dehydrogenase beta subunit	NS	NS
Q81IA3	123		Formate acetyltransferase	NS	NS
**Cofactors and vitamins metabolism**					
Q818X5	92	RibH	6,7-dimethyl-8-ribityllumazine synthase	NS	NS
Q818R9	405	Dxs	1-deoxy-D-xylulose-5-phosphate synthase	NS	NS
**Amino acid metabolism**					
Q817H8	189		Deblocking aminopeptidase	NS	NS
**Lipid and Fatty acid metabolism**					
Q81IN1	38		DAGKc domain-containing protein	NS	NS
**Pathogenesis**					
Q81I63	184		Microbial collagenase	NS	NS
Q81JC7	266		D-alanyl-D-alanine carboxypeptidase	NS	NS
**Defense mechanisms**					
Q81GN6	357	Kat	Catalase	−2,46	−1,93
**Transcription**					
Q81J66	312	MecB	Negative regulator of genetic competence	NS	NS
**Cys proteins in EEP**					
**Nucleotide metabolism**					
Q81IQ6	169	PurC	Phosphoribosylaminoimidazole-succinocarboxamide synthase	NS	NS
Q819S3	1034	CarB	Carbamoyl-phosphate synthase large chain	NS	NS
Q819S0	264	PyrB	Aspartate carbamoyltransferase	NS	NS
Q81FV5	38	DeoD	Purine nucleoside phosphorylase	NS	NS
**Translation**					
Q81IN3	185	GatA	Glutamyl-tRNA (Gln) amidotransferase subunit A	NS	NS
Q814C5	258	FusA	Elongation factor G	NS	NS
**Transcription**					
Q81J47	892	RpoC	DNA-directed RNA polymerase subunit beta	NS	NS
**Amino acid metabolism**					
Q816W9	315		Deblocking aminopeptidase	NS	NS
**Defense mechanisms**					
Q81IE5	110	TerD	Tellurium resistance protein	NS	NS
**Function unknown**					
Q81IG4	65		Thiamine_BP domain-containing protein	NS	NS
**Carbohydrate metabolism**					
Q81DX6	97	AdhA	Alcohol dehydrogenase	NS	NS
**Cys proteins in MEP**					
**Nucleotide metabolism**					
Q81J22	153	Adk	Adenylate kinase	NS	NS
Q81IS3	250	GuaA	GMP synthase	NS	NS
Q81IQ3	527	PurL	Phosphoribosyl formylglycinamidine synthase subunit	NS	NS
**Translation**					
Q814G7	15	RpsR	30S ribosomal protein S18	NS	NS
Q81J29	27	RpsZ	30S ribosomal protein S14 type Z	NS	NS
Q814C4	82	Tuf	Elongation factor Tu	NS	NS
**Transcription**					
Q81J47	818	RpoC	DNA-directed RNA polymerase subunit beta	NS	NS
Q81CE5	158	PpaC	Probable manganese-dependent inorganic pyrophosphatase	NS	NS
**Amino acid metabolism**					
Q81GG4	115	TrpA	Tryptophan synthase alpha chain	−3.08	−2.14
**Carbohydrate metabolism**					
Q818P6	194		Phosphoglycolate phosphatase	NS	NS
Q81DX6	91/255	AdhA	Alcohol dehydrogenase	NS	NS
**Cofactors and vitamins metabolism**					
Q818X3	115	Biob	Biotin synthase	NS	NS
**Protein folding**					
Q819C5	73		Peptidyl-prolyl cis-trans isomerase	NS	NS
**Cys proteins in LEP**					
**Amino acid metabolism**					
Q81GG5	378	TrpB	Tryptophan synthase beta chain	−2,24	−2,26
Q81GH0	168/65	TrpE	Anthranilate synthase component 1	−2,17	−2,17
Q81GG7	108	TrpC	Indole-3-glycerol phosphate synthase	−2,55	−2,26
Q81GG9	62	PabA (TrpGD)	Anthranilate synthase component II	−1,93	−1,39
Q814M2	364		O-acetylhomoserine sulfhydrylase	NS	NS
**Carbohydrate metabolism**					
Q819M9	262		Pyruvate carboxylase	NS	NS
Q81FZ7	124		Glycerate dehydrogenase	NS	NS
Q81A22	50	PorB	Pyruvate synthase subunit	NS	NS
Q81IA2	12	PflA	Pyruvate formate lyase activating enzyme	NS	NS
Q817F3	283	PfkA	ATP-dependent 6-phosphofructokinase	NS	NS
Q819D7	365	Ptsɪ	Phosphoenolpyruvate-protein phosphotransferase	NS	NS
**Cofactors and vitamins metabolism**					
Q81JC6	128/130	PdxS	Pyridoxal 5′-phosphate synthase lyase	NS	NS
Q816H9	71	MenB	1,4-dihydroxy-2-naphthoyl-CoA synthase	NS	NS
Q818W9	69	BioD	ATP-dependent dethiobiotin synthetase	NS	NS
**Transcription**					
Q81J18	265	RpoA	DNA-directed RNA polymerase subunit alpha	NS	NS
**Translation**					
Q814C4	138	Tuf	Elongation factor Tu	NS	NS
**Nucleotide metabolism**					
Q81JC8	327	GuaB	Inosine-5′-monophosphate dehydrogenase	NS	NS
**Defense mechanisms**					
Q81IK9	166	AhpC	Alkyl hydroperoxide reductase C	NS	NS
**Lipid and Fatty acid metabolism**					
Q814Y7	91	FabZ	3-hydroxyacyl-[acyl-carrier-protein] dehydratase	NS	NS
Q816B6	46/49	NfuA (YutI)	NifU protein	−1.9	−1,68
Q816D2	117		HESB protein	NS	NS
**Secondary metabolites biosynthesis, transport and catabolism**					
Q81DQ3	14	DhbC	Isochorismate synthase	−2.71	−3.06
Q81DQ1	67	DhbB	Isochorismatase	−2.55	−2.52
**Energy production and conversion**					
Q814U7	135		Nitrilotriacetate monooxygenase component B	NS	NS

Protein identifiers are underlined for proteins with differences in abundance; Cysteine positions are underlined for Cys-peptides detected with a higher proportion of RedCys at LEP; *FC* fold change; *NS* not significant

Several proteins were categorized in functional groups related to defense mechanisms. Superoxide dismutase SodA2 (Q814I6) contains a Cys residue (Cys^60^) identified as redox-sensitive at MEP, this protein was more abundant during LEP than during EEP (Table S2). The abundance of catalase (Kat, Q81GN6) increased during the LEP compared to both EEP and MEP, it harbors two redox-sensitive residues, Cys^357^, which are mainly detected as RedCys both at MEP and LEP; and Cys^458^, which is mainly detected as RedCys both at EEP and LEP. Thiol peroxidase (Tpx, Q817B8) contains two redox-sensitive Cys residues (Cys^60^ and Cys^94^), indifferently detected as RedCys and OxCys throughout growth. Finally, alkyl hydroperoxide reductase C (AhpC, Q81IK9) contains one Cys residue (Cys^166^), which was identified as redox-sensitive in the three growth stages, and mainly detected as RedCys at LEP.

Among the proteins categorized in the translation-related functional group, three are known to bind tRNA. These proteins harbored RedCys residues at EEP and MEP: Cys^170^ in PheT1 (Q812N7), Cys^124^ in IleS1 (Q819R4) and Cys^153^ in ArgS (Q814Q8). The elongation factor FusA (Q814C5) contains a Cys residue (Cys^258^) that was mainly detected as RedCys at EEP. The elongation factor Tuf (Q814C4) was associated with two redox-sensitive Cys residues (Cys^82^ and Cys^138^), which were mainly detected as RedCys at MEP and LEP, respectively.

Several proteins involved in nucleotide metabolism were identified in our study, these included PurD (Q81IP8), Adk (Q81J22) and GuaB (Q81JC8). Interestingly, the three Cys residues present in Inosine-5′-monophosphate dehydrogenase (GuaB) were found to be redox-sensitive at all three growth stages: Cys^308^, which contributes to the active site, indifferently detected as RedCys and OxCys throughout growth, whereas Cys^446^ was detected as RedCys at all three growth phases, and Cys^327^ was detected mainly as RedCys specifically at LEP.

Five proteins involved in amino acid metabolism were also identified. TrpA (Q81GG4), TrpB (Q81GG5), TrpC (Q81GG7), and PabA (Q81GG9). These proteins were more abundant in samples harvested at LEP compared to both EEP and MEP. Anthranilate synthase component I TrpE (Q81GH0) was more abundant at LEP than at MEP (Table 2).

In addition to these classes of proteins, we identified RedCys residues in iron-sulfur cluster proteins such as NifU (Q816B6), which is involved in the biogenesis of bacterial Fe/S proteins, and is thus responsible for several functions, including redox catalysis [34]. The abundance of NifU increased significantly during LEP compared to EEP and MEP.

Interestingly, based on Cys-peptides and spectral count analysis, we detected three proteins involved in the assembly of the bacillibactin siderophore during the LEP. These proteins, the isochorismate synthase DhbC (Q81DQ3), the isochorismatase DhbB (Q81DQ1) and the 2,3-dihydroxybenzoate-AMP ligase DhbE (Q81DQ2) were only detected in samples harvested during the LEP. In DhbE, Cys^473^ was indifferently detected as RedCys and OxCys. DhbB harbors two Cys residues, Cys^67^ and Cys^197^, indifferently detected as RedCys and OxCys. In contrast, Cys^14^ in DhbC was mainly detected as RedCys_._

## Discussion

Thiol redox modifications play important roles in regulating proteins that sustain bacterial growth. However, despite its importance, and mainly due to technical challenges, few studies have attempted to quantify changes in the bacterial thiol redox proteome during growth. The first hurdle relates to the extreme sensitivity of Cys residues to artefactual oxidation. For our study, we circumvented this problem by performing rapid quenching with TCA [35]. A second challenge is to use an efficient strategy to profile thiol redox modifications in the whole organism. We adopted a strategy without peptide enrichment steps to avoid introducing quantitative bias which could lead to deviations from physiological conditions, as we wished to conserve the situation in the cell at the time of harvesting as far as possible. To achieve our aims, we implemented a differential thiol trapping technique, the IDN strategy, which relies on irreversible modifications of reduced Cys residues by IAM, and oxidized Cys residues by NEM after DTT treatment [32, 36]. This strategy does not make it possible to characterize the OxCys forms, and to establish if they are part of an inter- or intra-polypeptide disulfide.

In any differential labeling strategy, the first alkylation – to block free thiols – must be highly efficient. Indeed, incomplete alkylation would increase the number of Cys available for NEM-labeling, leading to incorrect identification of oxidized residues [17]. In a previous study, Shakir et al. [25] used a sulfhydryl reactive dye (DyLight 550 maleimide) to estimate the proportion of free thiols after IAM labeling as a means to assess the efficiency of IAM alkylation. Here, we included a control sample (IN) to demonstrate and precisely quantify how efficiently IAM blocked free thiols. We were thus able to correct the data and obtain accurate quantifications of the NEMCys target. Through our approach, we identified 153 reactive Cys residues in *B. cereus* thiol proteome. This relatively low number of redox-target Cys may be explained by the overall low Cys content of proteins in *B. cereus* (less than 1%) [23]. Like the other facultative anaerobic Gram positive Firmicutes, *B. cereus* probably promotes cysteine exclusion from both its cytoplasmic and exported proteins due to their high sensitivity to reactive oxygen and nitrogen species (ROS and RNS) [37]. Indeed, any such sensitivity could pose problems for an organism that can survive in a wide variety of, sometimes hostile, environments. Another possibility is that by restricting our analysis to Cys residues identified in all three replicates at each stage of growth to ensure the data generated were reliable and reproducible, we may have excluded some redox-sensitive Cys residues. However, our results are of the same order of magnitude as those reported for *Caenorhabditis elegans*, for which fewer than 200 redox-sensitive Cys residues were identified during its lifespan [38].

Our study revealed that the number of detected reduced Cys residues increased as growth progressed. Extracellular ORP decreased at the same time, thus *B. cereus* growth under anaerobiosis is accompanied by a significant decrease in extracellular ORP, especially between the beginning and the end of exponential growth [29]. The ability to decrease extracellular ORP is common to many bacteria, and may be related to the consumption of oxidizing compounds or the production of reducing end-products, as reported for *E. coli* [39]. In *Lactococcus lactis* and *Listeria monocytogenes* the decrease in extracellular ORP has been shown to depend on the presence of reduced thiol groups present on proteins located at the bacterial cell surface [40, 41]. If the number of reduced thiol groups of these proteins increases as growth progresses, as in the case of the thiol groups in the proteins identified in this study, it could drive the reduction in extracellular ORP. However, this hypothesis will need to be proven through future research.

Overall, redox-sensitive Cys residues were mainly detected in their reduced forms, according to the reducing environment of cytoplasm [35], and the extracellular milieu in our condition. Only two Cys residues were detected more frequently as OxCys than as RedCys. In addition to being dependent of cellular conditions, the reactivity of Cys residues is partly controlled by the intrinsic properties of the sulfur atom and by the position of the Cys residue in the three-dimensional protein structure [42, 43]. Reactivity can also be modulated by proximity to a metal ion [44]. Interestingly, the two Cys residues, which were mainly detected as OxCys in our samples both belonged to alcohol dehydrogenase AdhA (Q81DX6). The predicted three-dimensional structure of the protein (data not shown) localizes these two residues, Cys^91^ and Cys^97^, near a Zn^2+^ binding site, which is assumed to promote oxidation [45].

Several redox-sensitive Cys-containing proteins identified in this study (Adk, PpaC, PurL, GuaB) were previously reported to undergo thiol modifications under oxidizing conditions in *Bacillus subtilis*, *Staphylococcus aureus, Corynebacterium glutamicum, and E. coli* [27, 28, 46, 47]. These reports suggest that these proteins could undergo specific redox regulation in response to variations in environmental conditions.

Our results showed that the high detection of RedCys residues at LEP was due to differences in abundance for 8 Cys-containing proteins (including 3 Cys-containing proteins that were detected exclusively at LEP) and the detection of 21 Cys-containing proteins with a higher number of RedCys at LEP without abundance variation. Therefore, the growth-phase-specificity of *B. cereus* thiol proteome may be explained both by a change of proteome composition and a distinct redox sensitivity of Cys residues in individual proteins [48]. The results of the Panther Gene Ontology analysis (http://geneontology.org/) illustrated the composition changes of *B. cereus* proteome (Figure S3). This analysis showed an enrichment of proteome in proteins involved in siderophore biosynthesis, and tryptophan biosynthesis at LEP, at the expense of proteins involved in deoxyribonucleotide biosynthesis. No proteome change characterizing the onset of sporulation has been detected, according to previous work [49].

Many abundant Cys-proteins, such as those involved in carbohydrate metabolism (PtsI and PfkA) and translation (FusA and Tuf), harbored cysteines displaying an increasing RedCys residues as growth progressed. These results suggest that these proteins may be redox-regulated to ensure efficient glucose uptake and protein biosynthesis over the course of growth. Moreover, redox regulation of PtsI and PfkA could potentially divert the metabolic flux toward post-translational modifications [50]. Interestingly, the modifications observed on Cys^82^ and Cys^138^ in the elongation factor Tuf at MEP, and LEP, respectively, could reflect two different sites of potential redox regulation during anaerobic growth. A growth-phase-dependent modification of Cys residues in Tuf could be the result of a growth-phase-dependent regulation network, as previously described [51, 52]. The redox-sensitive Cys residues in the Tuf protein were previously described to be S-bacillithiolated and S-mycothiolated in response to hypochlorite stress [28, 53]. In addition, Cys^82^ in Tuf was identified as a conserved S-bacillithiolated site among *Bacillus spp*. and *Staphylococcus carnosus* [54].

Our data showed that some redox-sensitive Cys-containing proteins were detected during LEP, due to their high abundance at this stage of growth, such as Kat, NifU, DhbC, and DhbB, which had at least one Cys detected mainly as RedCys during LEP. From a physiological point of view, these data support the entry of bacteria into the stationary phase [55, 56] in response to stress and nutrient starvation. The Fe-S-containing protein NifU is involved in sensing iron and superoxide levels [57], catalase is produced to deal with oxidative stress [58], and the enzymes involved in bacillibactin biosynthesis circumvent iron starvation [56]. To our knowledge, this is the first study to demonstrate the presence of reactive cysteine residues in DhbC and DhbB. Redox regulation of DhbB (Q81DQ1) remains uncharacterized, but might be important since this enzyme uses a thiolation mechanism to activate siderophore assembly [59].

## Conclusions

In summary, we presented an overview of the *B. cereus* ATCC 14579 thiol proteome, which is mainly defined by a subset of redox-sensitive Cys residues differing from one growth stage to another, and functionally related to growth-stage-dependent cellular events. This works represents an essential step for researchers in the fields of bacterial physiology toward understanding and predicting the effect of changing redox conditions.

## Methods

### Chemicals

Trichloroacetic acid (TCA), dithiothreitol (DTT), Trifluoroacetic acid (TFA), and iodoacetamide (IAM) were purchased from Sigma (St-Quentin-Fallavier, France). N-ethylmaleimide (NEM) and bicinchoninic acid assay (BCA) were purchased from Fisher Scientific (France). Trypsin and ProteaseMax reagent used for proteolysis were purchased from Promega (France).

### Regulated batch culture

*B. cereus* was grown in regulated batch conditions (pH 7, temperature = 37 °C, pO_2_ = 0%) in MOD medium supplemented with 30 mM glucose [6]. Briefly, *B. cereus* ATCC 14579 strain [60] was grown in batch cultures under anoxic conditions in a 3-L-capacity bioreactor (My-control, Applikon technology) equipped with a pH electrode (405-DPAS-SC-K8S/225, Mettler Toledo), a pO_2_ polarographic oxygen electrode (Z010023525, Applisens), and an AgCl ORP electrode (pT4805-SC-DPAS-K8S/225, Mettler Toledo). All batch culture experiments were conducted in 2 L MOD medium supplemented with 30 mM glucose, as described previously [29]. Continuous sparging of pure N_2_ (10 L/h) was maintained to generate anoxic conditions (pO_2_ = 0%). Cultures were stirred at 300 rpm, and temperature was set to 37 °C. The pH of the cell culture medium was maintained at 7 by adding 1 M HCl and 3 M KOH.

*B. cereus* cultures were performed in biological triplicates and the optical density (OD) at 600 nm was measured every 30 min. ORP values were corrected based on the value recorded by the reference electrode (+ 207 mV at 37 °C). The specific maximum growth rate (μ_max_) was determined using the Zwietering growth model [61].

### Protein extraction: quenching cellular thiol-disulfide exchange

Culture extracts were harvested anaerobically during the early-exponential growth phase (EEP) (at μ_max_), mid-exponential growth phase (MEP), and late-exponential growth phase (LEP) in vials containing 25% trichloroacetic acid (TCA). Extracts were incubated with TCA overnight at − 20 °C to maximize protein precipitation. Proteins were pelleted by centrifugation (13,800 x g, 15 min, 4 °C) and washed twice with ice-cold acetone to remove excess TCA. Supernatants were discarded, and protein pellets were air-dried.

### Differential thiol trapping method

Differential thiol labeling was performed as described previously [32, 62], with minor modifications. Briefly, protein extracts resuspended in denaturing buffer were divided between two tubes, labeled IDN, and IN (Fig. 2). Free cysteine residues were alkylated with 50 mM iodoacetamide (IAM), at room temperature in the dark for 1 h. Proteins were precipitated once again with 25% (w/v) TCA and washed twice with ice-cold acetone. Protein pellets were resuspended in the same buffer, and the IN samples were incubated with 150 mM N-ethylmaleimide (NEM). IDN samples were first reduced by adding 25 mM dithiothreitol (DTT), and incubating at room temperature for 1 h. Reduced proteins were TCA precipitated (25%), and resuspended before alkylation with 150 mM NEM.

The protein concentration of each sample was estimated using the bicinchoninic acid assay (BCA), according to the manufacturer’s protocol (Thermo-Fisher). A 90-μg aliquot of proteins was loaded onto a 12% SDS-PAGE for a short migration at 90 V (15–20 min). Gels were stained with imperial™ protein stain (Thermo-Fisher) for 1 h and destained with water. Protein bands were excised from gels. IDN and IN samples were digested with trypsin using the ProteaseMax surfactant (Promega) as described elsewhere [63] without further reduction/alkylation. For all samples, the resulting peptide mixtures were dissolved in 0.1% trifluoroacetic acid (TFA) prior to nano LC-MS/MS analyses.

### High-resolution tandem mass spectrometry

Peptides were identified using a Q-Exactive HF mass spectrometer (Thermo Scientific) coupled to an ultimate 3000 nano LC system (Thermo Scientific). The system was essentially operated as previously described [64] with minor modifications. The peptide mixtures (10 μL) were loaded and desalted online on a reverse-phase precolumn (Acclaim Pepmap 100 C18 5 μm bead size, 100-Å pore size, 5 mm × 300 μm, Thermo). They were then resolved on a nanoscale Acclaim Pepmap 100 C18 column (3 μm bead size, 100-Å pore size, 15 cm × 75 μm) at a flow rate of 200 nl/min using a 120-min gradient combining buffer B (0.1% HCOOH, 80% CH_3_CN) and buffer A (0.1% HCOOH, 100% H_2_O): 4–25% B in 100 min, followed by 25–40% B in 20 min. The mass spectrometer was operated in the Top20 data-dependent acquisition mode with full MS scans acquired from 350 to 1800 m/z at 60,000 resolution, and after each scan, selection of the 20 most abundant precursor ions for fragmentation and MS/MS acquisition at 15,000 resolution. An intensity threshold of 9 × 10^4^ was applied. A 10-s dynamic exclusion was used to increase the detection of low-abundance peptides. Only double- and triple-charged ions were selected for MS/MS analysis.

### MS/MS spectra interpretation

MS/MS spectra were queried against the theoretical *B. cereus* ATCC 14579 proteome (5216 sequences) with the Mascot Daemon software, version 2.6.1 (Matrix Science). The parameters were: full trypsin specificity, only 2+ and 3+ peptide charges, a mass tolerance of 5 ppm on the parent ion, a mass tolerance of 0.02 Da on the MS/MS ions, a maximum of two missed cleavages, no static modifications, and dynamic modifications were N-ethylmaleimide (C), Carbamidomethyl (C), Carbamyl (K), Oxidation (M), Deamidation (NQ). All peptide matches with a peptide score associated with a *p*-value of less than 0.05 were parsed using IRMa 1.31.1c software [65]. Proteins were considered valid when at least two different peptides were detected in the same sample. The proteomics datasets were submitted to the ProteomeXchange Consortium via the PRIDE partner repository [66], under dataset identifiers PXD022049 and 10.6019/PXD022049. [The dataset can be accessed with the Username: reviewer_pxd022049@ebi.ac.uk and Password: NFX3eY3n].

### Data analysis

All experiments were replicated three times. For each Cys residues, we compared the mean value of the number of reduced Cys residues with the mean value of the number of oxidized Cys residues. Statistical analysis was performed using the paired Student t-test, setting the threshold for statistical significance at *p* less than 0.05. For proteome profiling, data were normalized relative to the total spectral count. Protein abundances were compared between two growth stages, as previously described [67]. Proteins for which abundances changed by more than 1.5 were considered significant when the *p*-value was less than 0.05. Protein functions were assigned based on information from the Clusters of Orthologous Groups (COG) database [68].

## Supplementary Information

**Additional file 1: Table S1.** Non-redundant Cys-peptides identified in samples harvested at EEP, MEP, and LEP, in the IDNc dataset. **Table S2.** List of 117 Cys-proteins identified. From column Q to V: abundance levels of Cys-proteins (log2 values of spectral counts for each protein with the adjusted *p*-value).

**Additional file 2: Figure S3.** Changes in *B. cereus* proteome composition between EEP, MEP, and LEP obtained by Gene Ontology analysis of pathway enrichment.

## Data Availability

All data and materials are fully available without restriction. The proteomics datasets were submitted to the ProteomeXchange Consortium via the PRIDE partner repository [Project accession: PXD022049; Project DOI: 10.6019/PXD022049] and can be accessed at the URL [https://www.ebi.ac.uk/pride/login] with the Username: reviewer_pxd022049@ebi.ac.uk and Password: NFX3eY3n.
