# Supplementary material for: Redox proteomic study of Bacillus cereus thiol proteome during fermentative anaerobic growth
Source: BMC Genomics. 2021 Sep 7;22:648. doi: 10.1186/s12864-021-07962-y (PMC8425097; doi:10.1186/s12864-021-07962-y)

Figure S3: Changes in *Bacillus cereus* proteome composition between EEP, MEP, and LEP obtained by Gene Ontology analysis of pathway enrichment.


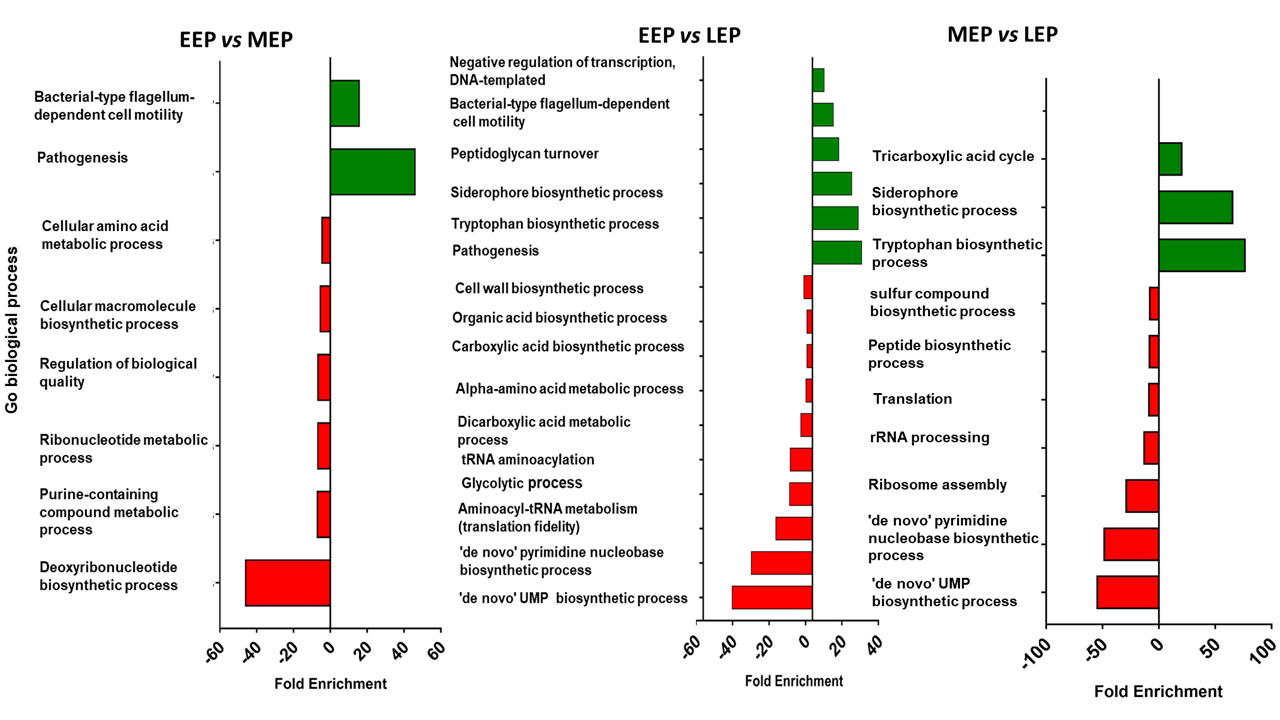

Supplement: Supplementary file 2 — Additional file 2: Figure S3. Changes in B. cereus proteome composition between EEP, MEP, and LEP obtained by Gene Ontology analysis of pathway enrichment. [file 12864_2021_7962_MOESM2_ESM.docx]
